# Natural Toxins for Use in Pest Management

**DOI:** 10.3390/toxins2081943

**Published:** 2010-07-29

**Authors:** Stephen O. Duke, Charles L. Cantrell, Kumudini M. Meepagala, David E. Wedge, Nurhayat Tabanca, Kevin K. Schrader

**Affiliations:** Natural Products Utilization, Agricultural Research Service, United States Department of Agriculture, P. O. Box 8048, University, MS 38677, USA; Email: charles.cantrell@ars.usda.gov (C.L.C.); Kumudini.Meepagala@ars.usda.gov (K.M.M.); david.wedge@ars.usda.gov (D.E.W.); Nur.tabanca@ars.usda.gov (N.T.); Kevin.schrader@ars.usda.gov (K.K.S.)

**Keywords:** algicide, fungicide, herbicide, insecticide, molluscicide, pesticide

## Abstract

Natural toxins are a source of new chemical classes of pesticides, as well as environmentally and toxicologically safer molecules than many of the currently used pesticides. Furthermore, they often have molecular target sites that are not exploited by currently marketed pesticides. There are highly successful products based on natural compounds in the major pesticide classes. These include the herbicide glufosinate (synthetic phosphinothricin), the spinosad insecticides, and the strobilurin fungicides. These and other examples of currently marketed natural product-based pesticides, as well as natural toxins that show promise as pesticides from our own research are discussed.

## 1. Introduction

In the developed world, pests are largely controlled by chemicals that are toxic to them. The vast majority of theses pesticides are synthetic compounds, some of which are based on natural toxins, and a few of which are synthetic versions of natural toxins. The pesticide industry has favored synthetic pesticides for several reasons, including: (1) the physicochemical properties of the natural pest-active compounds are often unsuitable for use as a pesticide; (2) natural toxins are often too structurally complex (e.g., multiple stereogenic centers) to be economical pesticides; (3) pesticide efficacy can often be improved by structural alteration and (4) intellectual property for synthetic compounds is often more easily obtained and defended. 

However, pesticide needs and the pesticide industry are changing. There is a growing requirement to produce more toxicologically and environmentally benign pesticides, and natural products often fill this need. There is a strong desire to use “greener” chemistry in pest management, especially in urban settings and in the production of edible horticultural crops. The cost of regulatory approval of some natural product pesticides is considerably less than that for synthetic pesticides. Pests have evolved resistance to many of the current pesticides, often by alterations in the molecular target site. Thus, pesticides with new molecular target sites and modes of action are needed. Natural pesticides often have molecular target sites that are not among those of synthetic pesticides (reviewed in [1]). Finally, organic food production is growing steadily in the developed world, and organic farmers will only accept natural pesticides. 

To be acceptable, pesticides must not have strong toxicity toward non-target organisms, especially humans. Yet, to be efficient, they must be highly toxic toward their intended targets. The mechanism of this type of selectivity is often the targeting of a molecular target site that is found only in the pest or, if in other organisms, is particularly vulnerable in the pest; e.g., an enzyme form that is significantly different from that of other organisms. 

This short review covers some of the natural toxins that are actually used in pest management, as well as a few others that have been patented or proposed as pesticides. Other reviews that cover this topic in more detail or from different perspectives are available [2,3,4]. 

## 2. Weed Management

### 2.1. Overview

Since the mid-twentieth century, synthetic herbicides have replaced other methods of weed management, greatly reducing the cost, while increasing the efficiency of weed control. Few herbicides and herbicide classes are based on natural toxins, despite considerable study of this topic and the many herbicide patents of natural toxins and their derivatives. There are a few exceptions. This subject has been previously reviewed in detail [5]. 

### 2.2. Phosphinothricin and Glufosinate

**Figure 1:**
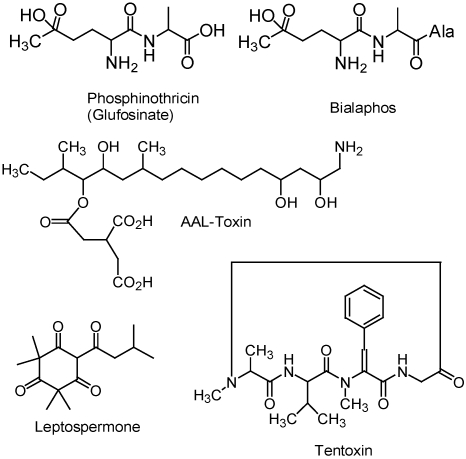
Structures of natural toxins that have been proposed as herbicides, are commercial herbicides, have been the structural basis for commercial herbicides.

The herbicide glufosinate is a racemic mixture of L- and D-phosphinothricin. Glufosinate is a non-selective, foliarly-applied herbicide that is considered to be one of the safest herbicides from a toxicological or environmental standpoint. L-phosphinothricin (butanoic acid, 2-amino-4-(hydroxymethylphosphinyl)-, (2*S*)-) (Figure 1), a potent and irreversible inhibitor of glutamine synthetase (GS), is produced by the soil microbe *Streptomyces hygroscopicus*. Inhibition of GS in green plants causes ammonia accumulation and rapid inhibition of photorespiration [5]. The effects of phosphinothricin on plants is too rapid to be due to reduced protein synthesis because of reductions in glutamine and other amino acids derived from glutamine. The high ammonium ion levels caused by phosphinothricin were thought to cause the rapid phytotoxic response. However, this view was not supported by the finding that the effects of the herbicide are ameliorated by supplying the plant with glutamine. Ammonium ion levels are not reduced by glutamate. Most of the phytotoxicity of inhibiting GS in C_3_ plants is apparently due to rapid inhibition of photorespiration, resulting in accumulation of glyoxylate in the chloroplast, which causes inhibition of ribulose bisphosphate carboxylase. When carbon fixation is stopped in the light, a series of events that ends with severe photodynamic damage occurs. 

Several other natural GS inhibitors are known, but none have all of the desirable traits of a herbicide possessed by phosphinothricin [6]. None of these compounds have been commercialized, other than the tripeptide bialaphos (4-[hydroxy(methyl)phosphinoyl]-L-homoalanyl-L-alanyl-L-alanine) (Figure 1), also produced by *Streptomyces hygroscopicus*. This is a very fermentation product sold on a limited scale for weed control in Japan. The market for this product is very small. The tripeptide has no activity on GS, but it is metabolically degraded to L-phosphinothricin in target plants. Thus, it is a protoxin.

*S. viridochromogenes* produces L-phosphinothricin and protects itself from the toxin with an enzyme that acylates the toxin, phosphinothricin acyl transferase (PAT). The acylated form of phosphinothricin is not phytotoxic. A slightly different form of the gene produced by *S. hygroscopus* encodes bialaphos resistance gene (*bar*) that also acylates phosphinothricin. These genes are used extensively in plant molecular biology as selectable markers, in that transformants can easily be selected with L-phosphinothrin. The use of glufosinate as a herbicide has been multiplied by the introduction of transgenic crops containing the *bar* gene. Glufosinate-resistant cotton, soybean, cotton, and maize are available to farmers in the U.S. [7]. Gene flow of the *bar* gene from experimental rice to commercial rice has been a problem. Glufosinate-resistant crops have not been as successful as glyphosate-resistant crops, but the evolution of glyphosate-resistant weeds is increasing their use. Glyphosate, the world’s most important herbicide, is structurally similar to glufosinate, but has an entirely different molecular target site [8]. At this time, there are no scientifically confirmed cases of evolved weed resistance to glufosinate. 

### 2.3. Phytotoxins from Microbes

Many other toxins from microbes have high levels of phytotoxicity [1], and many of these are patented as herbicides. For example, the cyclic tetrapeptide tentoxin (Figure 1) is both highly phytotoxic and highly selective, killing a number of important weeds at concentrations that have no effects on several important crops [9]. In other cases, the microbial toxin is too toxic to humans to consider as a herbicide. For example, AAL-toxin (Figure 1) is highly toxic to many weed species [10]. It is a close analog of the fumonisin mycotoxins, and like them is highly toxic to mammals by inhibition of ceremide synthase [11]. The relative plant/mammalian toxicity for AAL-toxin is higher for plants, whereas it is higher for mammals with fumonisins [12]. Extensive mammalian toxicity tests have not been done for most microbials toxins found to have activity against weeds. Phosphionthricin is the only microbial toxin that has been commercialized as a herbicide. 

### 2.4. Allelochemicals

Plants make many toxic substances. Those that are used by plants to combat other plant species are termed allelochemicals. No allelochemicals are used as herbicides, but one major class of herbicide, the triketones, were developed from a triketone allelochemical, leptospermone (Figure 1) [13]. Leptospermone, a constituent of the bottlebrush plant, is an inhibitor of hydroxyphenylpyruvate dioxygenase (HPPD) [14], an enzyme required for synthesis of tocopherols and plastoquinone in plants. Plastoquinone is an essential component of photosystem II of photosynthesis and is also a required cofactor for phytoene desaturase, a critical enzyme in carotenoid synthesis. The most common symptom following treatment of plants with HPPD inhibitors is white developing tissues, as lack of carotenoids results in bleaching of chlorophylls. 

No weeds have evolved resistance to HPPD inhibitors. Crops have been made resistant to this class of herbicides by genetic engineering, but these crops are not yet available. 

## 3. Insect Management

### 3.1. Overview

A cursory analysis of the impact of natural products on each of the various categories of pesticides reveals the remarkable contributions they have had on the agricultural insecticide market. Synthetic insecticides based on natural products have been around for decades and the impact is most evident when looking at the pyrethroids and neonicotinoids. More recently and thanks in part to the consumers demand for “organic” insecticides, there has been a huge surge in unmodified natural product insecticides, many of which are produced as microbial fermentation products. Such products and their acceptance by both consumers and farmers will continue to drive us towards even better unmodified natural product insecticides.

Sales of insecticides in a recent report further supports the trends mentioned above [15]. According to this report, five groups of insecticides (carbamates, neonicotinoids, pyrethroids, organophosphates, and natural products) accounted for over three-quarters of worldwide sales. Interestingly, three of these groups (neonicotinoids, pyrethroids, and natural products) are either completely natural product based (unmodified) or derived from natural products. Their combined worldwide sales accounted for 42.8% with the pyrethroids at 19.5%, neonicotinoids at 15.7%, and natural products at 7.6%. 

This section on natural product-based insecticides will focus primarily on unmodified natural products or natural product preparations, while those synthetic insecticides based on natural compounds will not be covered. Those compounds synthetically developed based on natural products, such as the pyrethroids and neonicotinoids, could be the subject of individual reviews and will not be discussed here. Natural product insecticides that are currently the most widely used and those that are newly introduced to the marketplace will be emphasized. 

### 3.2. Insecticides for Agricultural Use

#### 3.2.1. Spinosads

**Figure 2:**
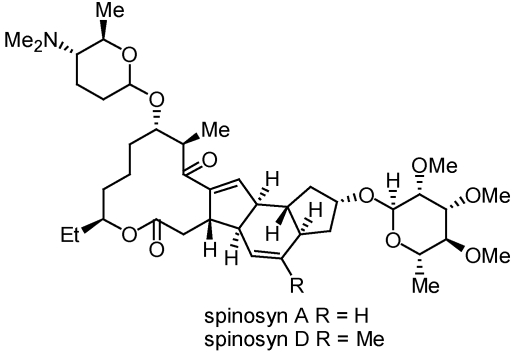
Structure of the major bioactive constituents isolated from the soil Actinomycete, *Saccharopolyspora spinosa*.

Originally isolated from the fermentation of the soil Actinomycete *Saccharopolyspora spinosa*, spinosad is a mixture of at least two major compounds, spinosyn A and spinosyn D (Figure 2) with spinosyn A being the major constituent. 

Spinosad appears to be effective by both ingestion and contact and causes excitation of the insect nervous system, leading to involuntary muscle contractions, prostration with tremors, and finally paralysis. These effects are consistent with the activation of nicotinic acetylcholine receptors by a mechanism that is clearly novel and unique among known insecticidal compounds [16,17,18]. 

Spinosad is approved for use as an organic insecticide. Spinosad is recommended for the control of a very wide range of caterpillars, leaf miners, thrips and foliage-feeding beetles. Spinosad products are quickly becoming a mainstay for not only organic farmers but also for conventional farmers. 

#### 3.2.2. Pyrethrum

**Figure 3:**
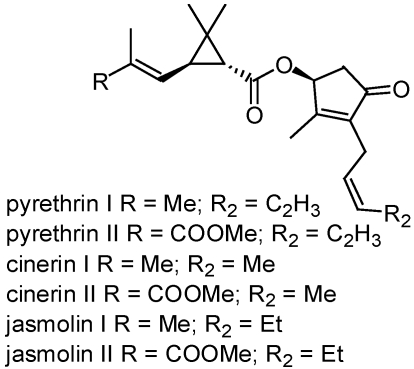
Structures of the six major constituents of pyrethrum.

Although we mentioned above that we would not discuss synthetic pyrethroids derived from natural products, we should briefly discuss pyrethrum, which refers to the oleoresin extracted from the dried flowers of *Tanacetum cinerariaefolium* (Asteraceae), formerly of the genus *Chrysanthemum*, and is the source of the pyrethrins, chrysanthemates and pyrethrates [1,19]. Products containing pyrethrum contain a mixture of at least six pyrethrin esters (Figure 3) [20]. This natural pyrethrum mixture of esters has highly unusual insecticidal properties and has been used safely and effectively for the past 160 years as a botanical insecticide around the world. It is fast acting and toxic to insects at very low doses, as well as degrading quickly in the environment due to its instability toward heat, light, and air. 

Many of the pyrethrum-containing products are approved for use in organic agriculture operations and are approved for controlling more than 40 insects on more than 200 different fruits and vegetables. The symptoms of pyrethrin poisoning are characterized by hyperexcitation, convulsions, seizures, and finally followed by death [21,22]. These symptoms are a result of the neurotoxic action, which block voltage-gated sodium channels in nerve axons. 

#### 3.2.3. Avermectins and milbemycins

**Figure 4:**
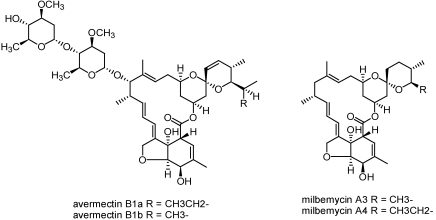
Structures of avermectins and milbemycins.

Discovered from *Streptomyces* sp. culture broths, the structurally similar avermectins and milbemycins, have had huge impacts in the field of animal health as agents against worms, ticks, flies and more recently on agricultural pests [23,24]. Abamectin, the active component of commercial avermectin products, is a mixture of >80% avermectin B_1a_ and <20% avermectin B_1b_ which are derived from the fermentation of the soil bacterium *Streptomyces avermitilis* [25,26] (Figure 4). Milbemectin, the predominant milbemectin component of commercial products, is a mixture of ≥70% milbemycin A_4_ and ≤30% milbemycin A_3_ which are derived from fermentation of the soil bacterium *Streptomyces hygroscopicus* subsp. *aureolacrimosus*. Milbemycins and avermectins have the same mode of action which is to potentiate glutamate and GABA gated chloride-channel opening [27]. The specific insects for which both milbemycin and avermectin products are approved for use are too numerous to mention here.

#### 3.2.4. Neem (Azadiracta indica) based products

**Figure 5:**
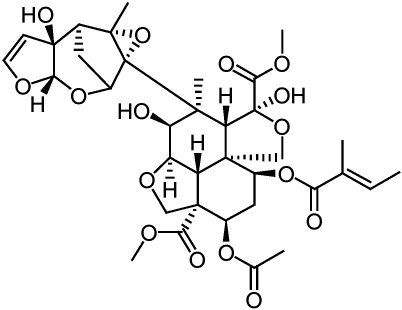
Structure of azadirachtin A.

The seeds from the Indian neem tree, *Azadirachta indica*, are the source of two types of neem-derived botanical insecticides; neem oil and medium polarity extracts. Neem seeds contain more numerous azadirachtin analogs, but the major form is azadirachtin A (Figure 5) and the remaining minor analogs likely contribute little to overall efficacy of the extracts [19]. 

Well known as a potent insect antifeedant, azadiractin A appears to work by blocking the synthesis and release of molting hormones (ecdysteroids) from the prothoracic gland [28]. Many neem/azadirachtin based products are approved for use as organic insecticides. An added advantage of neem oil based products is their ability to control fungal infections as well as a wide variety of both insect and mite pathogens. 

#### 3.2.5. Rotenone

**Figure 6:**
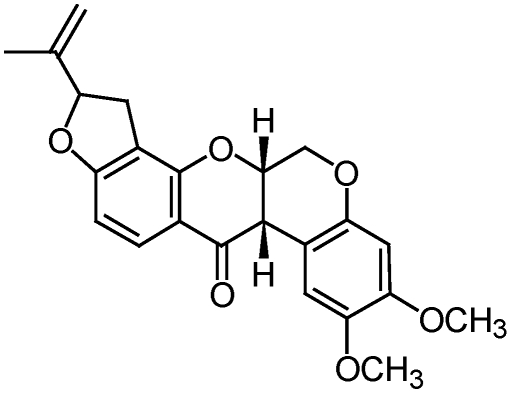
Structure of rotenone, a major constituent in rotenone insecticide preparations.

Like both neem and pyrethrum based products, rotenone insecticides have been in use for centuries. Products containing rotenone (Figure 6) are typically preparations from plant species of the genus *Derris* or *Lonchocarpus* (Leguminosae) with the majority from Cubé resin, a root extract of *Lonchocarpus utilis* and *Lonchocarpus urucu* [19]. Although rotenone is the major constituent in Cubé resin and hence in rotenone products, the active ingredients deguelin, rotenolone, and tephrosin are also present [29]. Rotenone based products are approved for use as organic insecticides under many trade names and most are sold as blends containing both rotenone and pyrethrum extracts. 

#### 3.2.6. Sabadilla

**Figure 7:**
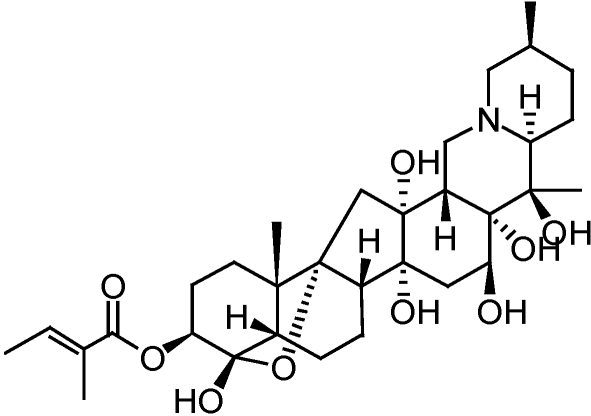
Structure of cevadine from sabadilla derived extracts.

Sabadilla-based products are derived from the seeds of plants from the genus *Schoenocaulon* and is predominantly from the sabadilla lily (*Schoenocaulon officinale*). The activity of sabadilla preparations is primarily due to the alkaloids cevadine and veratridine which typically exist in a 2:1 ratio and are collectively referred to as veratrine [30] (Figure 7). 

The mode of action of sabadilla alkaloids appears to be similar to that of the pyrethrins in that they work on voltage-sensitive sodium channels. Sabadilla is approved for usein the USA as an organic insecticide, as well as for other uses, by the Organic Materials Review Institute (OMRI). 

#### 3.2.7. Additional products and preparations

Aqueous tobacco (*Nicotiana tabacum*, *N. glauca or N. rustica*) extracts containing the alkaloid nicotine have long been used to control crop insect pathogens [31,32]. Nicotine is well known to exert its insecticidal effect by interacting with nicotinic acetylcholine receptors. The insecticide usually is marketed as a liquid concentrate of nicotine sulfate.

Plant essential oils and seed pressed oils make up a significant part of the market share for natural product based insecticides according to OMRI. Those essential oils that appear to be used in these products quite often include rosemary oil, thyme oil, and eugenol and/or clove oil. Many of these oil based products also possess herbicidal activity. 

Capsaicin based products are produced from plants of the genus *Capsicum* and are often derived from hot chili peppers (*Capsicum frutescens*). Products typically contain 3% capsaicin and are obtained by grinding the dry, ripe peppers and extracting the powder to obtain a resin after evaporation of the solvent. 

### 3.3. Insecticides for Urban Use

Termites and mosquitoes are two major urban pests that are important in the U.S. We will concentrate on these insects, as these are two of the most important urban insect targets of insecticides.

#### 3.3.1. Termites

Among the termite species, the Formosan subterranean termite (FST), *Coptotermes formosanus* Shiraki (Isoptera: Rhinotermitidae) is highly aggressive and destructive to timber and wood structures in the USA. This termite, a native of East Asia, most likely invaded North America as a result of disembarkation of wooden military cargo at the port of New Orleans and other coastal ports that arrived from Asia during and after World War II [33]. Since its first invasion of North America, this pest has flourished rapidly, spreading over other southeastern states and California. Devastation caused by *C. formosanus* in North America has been estimated to be one to two billion dollars a year. In the city of New Orleans alone, the control and repair costs due to the FST is estimated at $300 million annually. Over the past two decades, organo-chlorines and organo-phosphates, the two prominent classes of termite control agents, have been banned due to environmental and human health concerns. 

At the present time, synthetic pyrethroids (permethrin and cypermethrin), phenylpyrazoles (e.g., fipronil), chloronicotinyls (imidacloprid), and pyrroles (chlorfenapyr) are commercially available as termite control agents. Mammalian toxicity, non target toxicity and seeping of these compounds into ground water are some of the drawbacks associated with these synthetic termiticides. Availability and use of creosote and chromated copper aresenates as wood preservatives have also been limited due to environmental and health concerns. Therefore, a strong desire exists to replace these products with ‘greener’ termiticides. There are no completely natural product termiticides on the market, although all synthetic pyrethroids are derived from natural products. 

**Figure 8:**
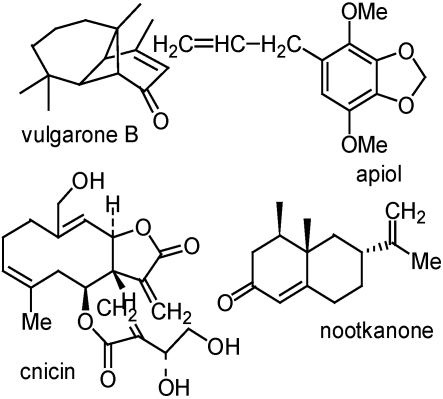
Some plant-derived compouds with termiticidal activity.

Plants are a good potential source of such compounds, since many plant species have a defense mechanism and produce secondary metabolites in order to survive in the ecosystem. The plant families Asteraceae [34,35], Milliaceae [36,37,38] and Apiaceae [39,40] are known to have insecticidal constituents. Vulgarone B (isolated from *Artemisia douglasiana*; Asteraceae), apiol (isolated from *Ligusticum hultenii*; Apiaceae), and cnicin (isolated from *Centaurea maculosa*; Asteraceae) cause significant mortality to Formosan subterranean termites in laboratory bioassays [41] (Figure 8). Vulgarone B, apiol, and cnicin are present in high levels in the plants from which they have been isolated and also possess other biological activities such as phytotoxic and antifungal properties suggesting the ecological importance of these secondary metabolites in these plants [42]. This is a reflection of the importance of plant secondary metabolites as natural defense mechanisms. Nootkatone, (Figure 8), a sesquiterpene ketone is from vetiver (*Vetiveria zizanioides*) oil has strong termiticide repellent and toxicant activities to Formosan subterranean termites [43]. 

#### 3.3.2. Mosquitoes

**Figure 9:**
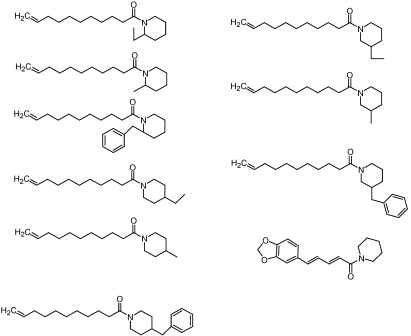
Chemical structures of the derivatives of 1-undec-10-enoyl-piperidines and piperine.

The yellow fever mosquito, *Aedes aegypti* (L.), is considered the primary vector for both dengue and yellow fever. Application of insecticides is one of the major methods to control this medically important insect pest. However, few new insecticides have been developed for mosquito control. As part of our effort to discover new insecticides for mosquito control, piperidine was used as a lead compound for further optimization. Piperine, (*E*,*E*)-1-piperoyl-piperidine, is the major constituent in *Piper nigrum* fruit. Amides of some piperidines containing the 1-undec-10-enoyl moiety have been synthesized and tested against female adults of *A. aegypti* (L.) [44] (Figure 9). On the basis of 24 h LD_50_ values after topical application, the most toxic compound was 2-ethyl-1-undec-10-enoyl-piperidine (LD_50_ = 0.80 μg per mosquito). Piperine [(*E*,*E*)-1-piperoyl-piperidine], a natural product from black pepper (*Piper nigrum*) that has insecticidal activity, was less toxic (LD_50_ = 8.13 μg per mosquito) than all the tested ethyl- or methyl- derivatives of 1-undec-10-enoyl-piperidines. The toxicities of 1-undec-10-enoyl-piperidines were significantly decreased when a benzyl moiety was added to the carbon of the piperidine ring, regardless the carbon position to which it was attached. Thus, substituted piperidine derivatives could be further developed as insecticides for adult mosquitoes. 

**Figure 10:**
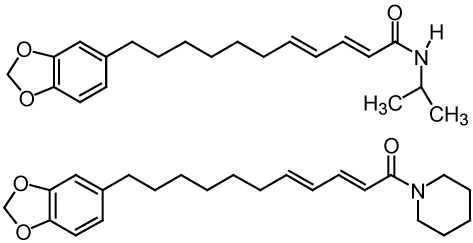
Amides with lavicide activity.

Amides are known to posses insecticidal properties. Several amides based on piperine have shown high larvicidal activity in laboratory bioassays when compared to indoxacarb, a commercial insecticide (Figure 10) [45]. The isobutyl amide derivative showed 100% mortality of mosquito larvae at 2 ppm where as the piperidine analog showed 80% mortality. At 2 ppm indoxacarb, isobutyl derivative and piperidine derivative showed 40%, 80%, and 20% mortalities, respectively. 

## 4. Mollusc Management

Certain molluscs (mollusks) can be a threat to agriculture and to public health as a disease vector. They are in the mollusca phylum ranging from tiny snails, clams, abalone, squid, cuttlefish and octopus. Molluscs are used as ornamental animals and a food delicacy and good protein source in many countries. They can be found in gardens, ponds, deserts and oceans. Molluscs that live in ponds and oceans have gills and form a paraphyletic group, and the land snails with lungs form the pulmonata group. Snails without shells are called slugs. Most molluscs are herbivores, but some species are omnivores. 

In the literature there are many natural compounds with diverse chemical structures that are molluscicidal. In order to search for natural product-based molluscicides one of the important factors is the cost and availability, as many of the mollusc-borne diseases occur in the underdeveloped or developing countries. Synthetic molluscicides are expensive, and many of them are non-biodegradable and persist for a long time in the environment, causing damage to non-target species. Thus, it is important to look into traditional folk medicine and ethnopharmacological information in order to search for natural product-based molluscicides. 

Development of extracts of the berries of endod (*Phytolaca dodecandra*) as a mollusicide to control schistosomiasis has been very successful in Ethiopia [46]. The active constituents have been isolated and identified as saponins [47]. *Lonicera nigra*, *Hedera helix*, *Cornus florida*, *Asparagus curillus* are among some other plants that have been investigated for molluscicidal saponins [47]. *Hedera helix* contains a hederagenine glycoside with LC_100_ at 3 ppm against *Biomphalaria glabrata* snails. 

Catfish (*Ictalurus punctatus*) is one of the major farm-raised fish in the U.S. The ram’s horn snail (*Planobdella trivolvis*) is an intermediate host for the digenetic trematode (*Bolbophorus confusus*) that has been discovered to be a significant problem in commercial channel catfish production ponds in the southern U.S. [48]. The life cycle of this parasitic trematode involves the snail, channel catfish, and the American white pelican (*Pelecanus erythrorhynchos*). At this time there is no cure or a treatment for the infected fish. One approach to eradicate or control this problem is to interrupt the life cycle of the parasitic trematodes by eliminating the snails, which are essential to the life cycle. Application of lime and copper sulfate around the catfish ponds are common practices to reduce snail populations. 

Vulgarone B (Figure 8), isolated from the steam distillate of the aerial parts of the plant *Artemisia douglasiana* (Asteraceae), has been found to be active towards ram’s horns nails with an LC_50_ of *ca* 24 µM [49]. This compound caused severe hemolysis associated with lethal activity to the snails at concentrations that were not toxic to channel catfish. 2Z,8Z-matricaria methyl ester isolated from *Erigeron speciosus*, a plant in the Asteraceae family has also shown molluscicidal activity against *P. trivolvis* with a LC_50_ of 50 µM [50]. In the laboratory experiments Yucca extract at 10 ppm caused 100% mortality of *P. trivolvis* [51] Hederagenin-3-*O*-β-D-glucopyranoside, isolated from English Ivy (*Hedera helix*), has good activity against *P. trivolvis*, with an LC_50_ of 30 µM [51].

The golden apple snail (GAS), *Pomacea canaliculata* (Lamarck), is a major pest of rice in all rice-growing countries, where it was either intentionally or accidentally introduced [52]. It is native to South America and was introduced to Asia as a protein source and a source of income for farmers in the rural, underdeveloped impoverished areas. In the Philippines, the government promoted GAS production as a national livelihood program to increase the protein intake of low-income Filipino rice farmers and as an additional source of their income. When it was found that the snail was a vector for a parasite, GAS snail farmers abandoned their cultures, and the snails were disposed of without precautions. GAS soon invaded the rice fields, which were ideal habitats with abundant food supply. GAS fed mainly on rice seedlings causing heavy losses in rice yields. In the United States, GAS is a problem in taro plantations in Hawaii.

Cost effective, target specific, and environmentally friendly molluscicides are in need due to the economic burden and undesirable effects of currently available commercial molluscicides (niclosamide and metaldehyde). Vulgarone B (Figure 8) is a potential molluscicide with a LC_50_ of about 30 μM at 24 h for GAS [52]. In the same bioassay, the standard commercial molluscicide, metaldehyde, also had an LC_50_ of about 30 μM. This corresponds to about 6.5 mg/L and 4.4 mg/L of the vulgarone B and metaldehyde, respectively. Vulgarone B caused mortality more quickly than metaldehyde. The concentrations needed for 100% mortality at 24 h were about 75 and 200 μM, respectively, for vulgarone B and metaldehyde. In practical terms, a rice farmer who consumes about 250 liters of water for spraying one hectare will require 4.8 g of vulgarone B for GAS control. There was no toxicity to 14-day-old rice plants 10 days after treatment at sprayed concentrations of vulgarone B that cause complete or nearly complete mortality of GAS. We also found in field and laboratory experiments that vulgarone B has a potential as a molluscicide in taro paddies in Hawaii [53]. 

## 5. Algae Management

In various freshwater ecosystems around the world, certain species of cyanobacteria (blue-green algae) are highly undesirable due to their production of toxins or odorous compounds. For example, in channel catfish (*Ictalurus punctatus*) aquaculture ponds in the southeastern United States, certain species of cyanobacteria produce the “earthy” and “musty” monoterpene metabolites geosmin and 2-methylisoborneol (MIB), respectively. These compounds rapidly absorb across the gills of the catfish and accumulate in the flesh, thereby rendering them unpalatable and unmarketable.

The approach used by most catfish producers in the southeastern US to mitigate earthy/musty off-flavor problems is the application of algicides to ponds to reduce the abundance of odor-producing cyanobacteria. The three types of algicides currently approved by the United States Environmental Protection Agency (USEPA) for use in aquaculture ponds are copper based-products (e.g., chelated-copper compounds, copper sulfate), diuron [*N*’-(3,4-dichlorophenyl)-*N*,*N*-dimethylurea], and sodium carbonate peroxyhydrate (SCP)-based products which release sodium carbonate and hydrogen peroxide upon dissolution. However, these compounds possess several negative attributes. The first two types of compounds have high environmental persistence while all three types have broad-spectrum toxicity towards phytoplankton. Also, an efficacy study of an SCP-formulated product did not reduce the abundance of odor-producing cyanobacteria in catfish ponds [54]. 

Until recently, very limited research had been performed to discover alternative natural and natural product-based algicides with greater selective toxicity towards undesirable types of cyanobacteria. 

**Figure 11:**
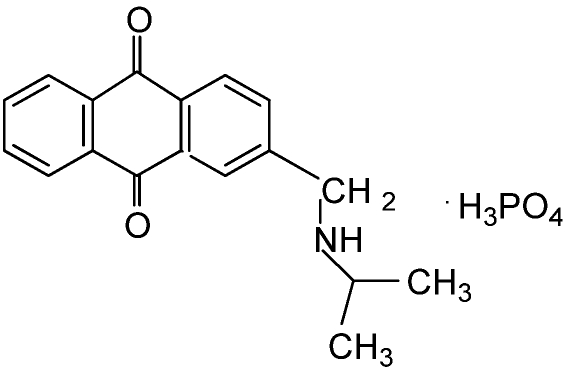
Water-soluble derivative of anthraquinone, referred to as anthraquinone-59.

A rapid bioassay developed by Schrader *et al.* [55] has been used to discover natural compounds from plants and marine organisms for potential use as selective algicides. Good selective activity of natural compounds in the laboratory does not always translate into good activity under field conditions. In the case of ferulic acid [56], the reduction in activity was due to a short half-life, a common problem with many natural compounds. One of the most promising compounds discovered is 9,10-anthraquinone [57]. Because 9,10-anthraquinone is relatively insoluble in water, the chemical structure of 9,10-anthraquinone was modified to impart water solubility [58]. Efficacy testing of two of these water-soluble derivatives of 9,10-anthraquinone found them to be effective in reducing the abundance of odor-producing cyanobacteria and MIB levels in catfish ponds and to be much less persistent than diuron in pond water [59]. In addition, one of the more promising anthraquinone analogs, anthraquinone-59 (Figure 11), has undergone toxicological evaluation which determined a “safety” margin of at least one order of magnitude between the concentration effective in reducing the abundance of odor-producing cyanobacteria in catfish ponds and the 96 h LC50 for channel catfish [60].

**Figure 12:**
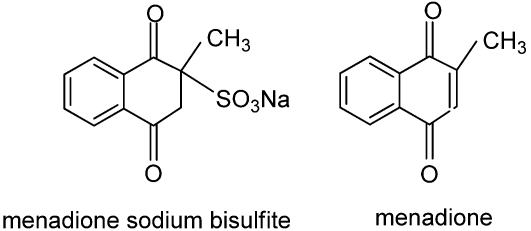
Chemical structures of quinone-based algicidal compounds.

SeaKleen^®^, a commercial biocide used to treat the ballast water of ships to prevent the spread of pest organisms such as algae [61], is another quinone-related natural compound determined to be effective in reducing the abundance of odor-producing cyanobacteria at application rates of 1.3 mg/L [62]. SeaKleen^®^ consists of menadione sodium bisulfite, a water-soluble form of menadione that is also known as vitamin K_3_ or 2-methyl-1,4-naphthoquinone (Figure 12). Any future USEPA approval of quinone-based algicides in catfish production ponds will require additional research including determination of the environmental fate of the quinone-based compound and any accumulation of the quinone-based compound in the flesh of the catfish. 

## 6. Plant Pathogen Management

Natural compounds for controlling plant pathogens have been reviewed before [63,64,65]. We will discuss only those compounds that are directly fungicidal or bactericidal. We will not cover elicitors that induce host plant defenses such as systemic acquired resistance, as these compounds and preparations cannot be considered toxins. Nor will we discuss mixtures, crude extracts, or essential oils because the toxins in these are poorly understood in terms of their fungicidal activities. 

**Figure 13:**
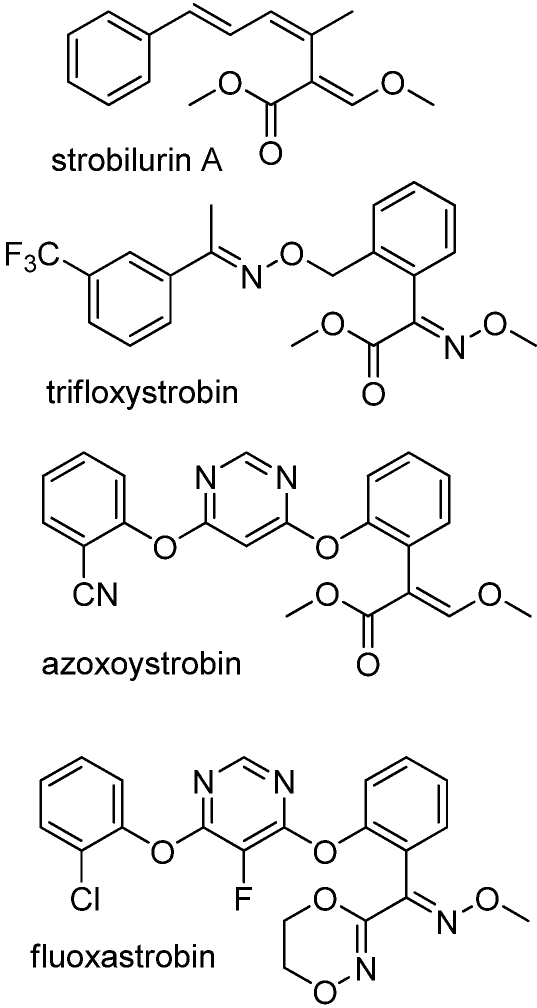
Natural and synthetic strobilurin fungicides.

The most successful commercial herbicides based on a natural product are the strobilurins. The synthetic fungicides (e.g., trifloxystrobiin, azoxystrobin, and fluoxastrobin) (Figure 13) are based on the structure of a natural class of compounds, the strobilurins, such as stobilurin A (Figure 13) from basidiomyces growing on decaying wood [66]. This class of compunds provided a new mode of action among commercial fungicides—inhibition of respiration at the complex III: cytochrome bc1 site. Unfortunately, resistance has already evolved to this class of fungicides in some plant pathogens in certain geographical areas. The strobilurin-based fungicides are the only major class of fungicides base on a natural product.

A number of antibiotics from microbes are used to a limited extent as fungicides, mainly in Japan [1]. These fermentation products include streptomycin, blasicydin S, kasugamycin, mildiomycin, natamycin, oxytetracyline, polyoxin B, polyoxorim, oudemanisin, and validamycin. To our knowledge, streptomycin is the only pharmaceutical also used as a pesticide.

Cinnamaldehyde, a plant product found in several plant species, is synthesized and sold as a fungicide. Its mode of action is through inhibition of fungal cell wall formation by inhibition of chitin synthesis [67,68].

**Figure 14:**
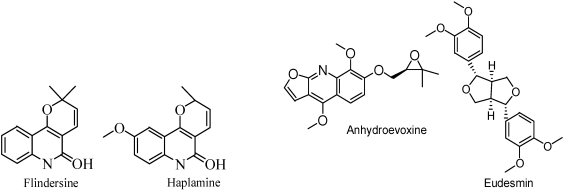
Fungicidal compounds isolated from *H. sieversii*.

**Figure 15:**
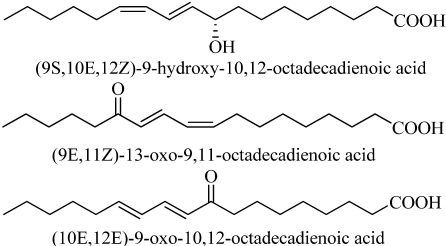
Antifungal oxylipins from the mushroom *Gomphus floccosus.*

**Figure 16:**
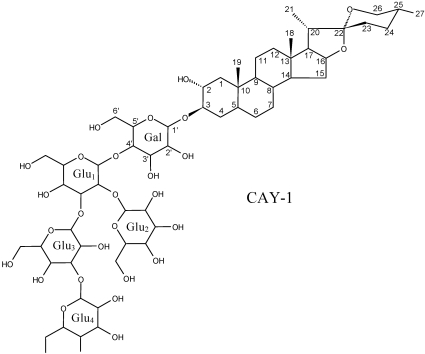
CAY-1, a fungicidal saponin from *Capsicum frutescens*.

Our laboratory has a research program to discover natural compounds that can be used as agricultural fungicides. Two such compounds from plant sources, sampangine from the root bark of the African tree *Cleistopholis patens* [69] and coruscanone A and B from *Piper coruscans* [70], have been patented. These compounds were originally tested as medicinal fungicides. Many other compounds from a variety of organisms have been found to have activity against fungal plant pathogens. These include several alkaloids from *Haplophyllum sieversii* [71] (Figure 14), several unusual fatty acids from the basidiomycete *Coptotermes formosanus* [72] (Figure 15), several thiophenes from the Mediterranean plant *Echinops ritro* (Asteraceae) [73], and a steroidal saponin from the cayenne pepper (*Capsicum frutescens*) [74] (Figure 16). 

## 7. Conclusions

The growing need for new pesticides with new molecular target sites, the desire for ‘greener’ pest management chemicals, and the improved ease of discovery of bioactive toxins is increasing the interest in natural products, many of which can be considered toxins, as pesticides. We have focused on the relatively small number of natural toxin or natural toxin-based products that are already available, as well as a few promising leads from our own research. These products represent only a small fraction of such compounds that have been patented for pesticide use, and a minute fraction of the toxins that have been reported to have potential utility as pesticides. We expect that many more natural toxin-based pesticides will become available in coming years.
